# How does study quality affect the results of a diagnostic meta-analysis?

**DOI:** 10.1186/1471-2288-5-20

**Published:** 2005-06-08

**Authors:** Marie E Westwood, Penny F Whiting, Jos Kleijnen

**Affiliations:** 1 Centre for Reviews and Dissemination, University of York, UK

## Abstract

**Background:**

The use of systematic literature review to inform evidence based practice in diagnostics is rapidly expanding. Although the primary diagnostic literature is extensive, studies are often of low methodological quality or poorly reported. There has been no rigorously evaluated, evidence based tool to assess the methodological quality of diagnostic studies.

The primary objective of this study was to determine the extent to which variations in the quality of primary studies impact the results of a diagnostic meta-analysis and whether this differs with diagnostic test type. A secondary objective was to contribute to the evaluation of QUADAS, an evidence-based tool for the assessment of quality in diagnostic accuracy studies.

**Methods:**

This study was conducted as part of large systematic review of tests used in the diagnosis and further investigation of urinary tract infection (UTI) in children. All studies included in this review were assessed using QUADAS, an evidence-based tool for the assessment of quality in systematic reviews of diagnostic accuracy studies. The impact of individual components of QUADAS on a summary measure of diagnostic accuracy was investigated using regression analysis. The review divided the diagnosis and further investigation of UTI into the following three clinical stages: diagnosis of UTI, localisation of infection, and further investigation of the UTI. Each stage used different types of diagnostic test, which were considered to involve different quality concerns.

**Results:**

Many of the studies included in our review were poorly reported. The proportion of QUADAS items fulfilled was similar for studies in different sections of the review. However, as might be expected, the individual items fulfilled differed between the three clinical stages. Regression analysis found that different items showed a strong association with test performance for the different tests evaluated. These differences were observed both within and between the three clinical stages assessed by the review. The results of regression analyses were also affected by whether or not a weighting (by sample size) was applied. Our analysis was severely limited by the completeness of reporting and the differences between the index tests evaluated and the reference standards used to confirm diagnoses in the primary studies. Few tests were evaluated by sufficient studies to allow meaningful use of meta-analytic pooling and investigation of heterogeneity. This meant that further analysis to investigate heterogeneity could only be undertaken using a subset of studies, and that the findings are open to various interpretations.

**Conclusion:**

Further work is needed to investigate the influence of methodological quality on the results of diagnostic meta-analyses. Large data sets of well-reported primary studies are needed to address this question. Without significant improvements in the completeness of reporting of primary studies, progress in this area will be limited.

## Background

The use of systematic literature review to inform evidence-based practice in diagnostics is rapidly expanding. Although the primary diagnostic literature is extensive, there remain a number of problems for systematic reviews of diagnostic tests. Appropriate methods for rigorous evaluation of diagnostic technologies have been well established [1-5]. However, available studies have generally been poorly designed and reported [6-8]. Similarly, although a number of quality checklists for diagnostic accuracy studies have been proposed [9] and there is growing evidence on the effects of bias in such studies [10], there has been no rigorously evaluated, evidence-based quality assessment tool for diagnostic studies.

The objective of this study was to investigate the impact of quality on the results of a diagnostic meta-analysis, using regression analysis. A large diagnostic systematic review was required to enable the use of regression analysis to investigate the impact of components of quality upon results.

We have recently completed a systematic review, which aimed to determine the most appropriate pathway for the diagnosis and further investigation of UTI in children [11]. It included an assessment of the accuracy of tests for three different clinical stages of UTI: the diagnosis UTI, localisation of infection, and further investigation of patients with confirmed UTI. The nature of the tests included in these three clinical sections of this review differed. Tests used to diagnose UTI were generally laboratory-based or near-patient methods, with relatively objective interpretation of results, e.g. dipstick tests and microscopy. By contrast, tests used to investigate confirmed UTI mainly utilised imaging technologies which are largely subjective in their interpretation, and where diagnostic thresholds are difficult to define. Tests used to localise infection spanned both categories. We hypothesised that the components of methodological quality affecting results were likely to differ between the three sections of the review. Such potential differences may indicate a need for topic-specific checklists for the assessment of quality in diagnostic studies.

A secondary aim of this study was to contribute to the evaluation of QUADAS, an evidence-based tool for the assessment of the quality of diagnostic accuracy studies that was specifically developed for use in systematic reviews of diagnostic tests [12], by investigating the importance of specific QUADAS items.

## Methods

We used QUADAS [12] (Table 1) to assess the quality of primary studies included in the review. Items were rated as 'yes', 'no', or 'unclear'. We examined differences in the individual QUADAS items fulfilled, as well as their impact on test performance. The review divided the diagnosis and further investigation of UTI into the following three clinical stages: diagnosis of UTI, localisation of infection, and further investigation of the UTI. Each stage used different types of diagnostic test, which were considered to involve different quality concerns.

**Table 1:** QUADAS

**Item #**	**Description**
1.	Was the spectrum of patients representative of the patients who will receive the test in practice?
2.	Were selection criteria clearly described?
3.	Is the reference standard likely to correctly classify the target condition?
4.	Is the time period between reference standard and index test short enough to be reasonably sure that the target condition did not change between the two tests? (disease progression bias)
5.	Did the whole sample or a random selection of the sample, receive verification using a reference standard of diagnosis? (partial verification bias)
6.	Did patients receive the same reference standard regardless of the index test result? (differential verification bias)
7.	Was the reference standard independent of the index test (i.e. the index test did not form part of the reference standard)? (incorporation bias)
8.	Was the execution of the index test described in sufficient detail to permit replication of the test?
9.	Was the execution of the reference standard described in sufficient detail to permit its replication?
10.	Were the index test results interpreted without knowledge of the results of the reference standard? (test review bias)
11.	Were the reference standard results interpreted without knowledge of the results of the index test? (diagnostic review bias)
12.	Were the same clinical data available when test results were interpreted as would be available when the test is used in practice? (clinical review bias)
13.	Were uninterpretable/ intermediate test results reported?
14.	Were withdrawals from the study explained?

We analysed results grouped by clinical stage. Within these groups, we pooled studies of similar tests or test combinations where sufficient data were available and where pooling was clinically meaningful. (Table 2) The minimum number of studies that we required for regression analysis was ten. This choice was made based on published guidance [13,14].

**Table 2:** Tests included/excluded in the regression analysis

**Tests included in the regression analysis (number of studies)**	**Tests for which there were insufficient studies to permit regression analysis (number of studies)**
**Diagnosis**	

*Dipstick*: nitrite (23), LE (14), nitrite or LE positive (15)	*Clinical history *(6)
*Microscopy*: pyuria (28), bacteriuria (22)	*Dipstick*: nitrite and LE positive (9), glucose (4), protein (2), blood (1), protein and LE positive (1), combinations of 3 dipstick tests (5)
	*Microscopy*: pyuria or bacteriuria (8), pyuria and bacteriuria (8)
	*Culture: *standard (1), dipslide (1)
	*Combinations of different tests *(10)

**Localisation**	

*Ultrasound *(20)	*Clinical history *(5)
	*Laboratory based tests *(16)
	*Imaging techniques: *MCUG (7), MRI (1), CT (1), IVP (4), cystography (2), scintigraphy (3)

**Further investigation**	

*Detection of reflux: *Ultrasound (28): standard (11), contrast enhanced (17)	*Detection of reflux: *IVP (4), voiding radionuclide cystography (3), NAG/creatinine ratio (1), scintigraphy, (3), risk scoring system (1)
	*Prediction of scarring: *ultrasound (2), IVP (1), non-invasive indicators (1), MCUG (2)
	*Detection of scarring*: IVP (4), static scintigraphy (7), dynamic scintigraphy (2), MCUG (4), cystography (1), MRI (1), US and MCUG (1)

We estimated summary receiver operator characteristic (SROC) curves using the following equation [15]:



a and b were estimated by regressing D against S for each study:

D = a + bS

D = {logit (sensitivity) - logit (1-specificity)} = log diagnostic odds ratio (DOR)

S = {logit (sensitivity) + logit (1-specificity)}

We used both weighted and unweighted models. For the weighted model we weighted on sample size. We chose to weight on sample size rather than inverse variance, a method sometimes used in this type of analysis, as we believe that weighting on the inverse variance can produce biased results. The reason for this bias is that the DOR is associated with its variance and so large DORs will inevitably have large variances, which will be reflected in the weightings.

We assessed between study heterogeneity through visual examination of forest plots and statistically using the Q statistic [16]. Where sufficient data were available, we used regression analysis to investigate whether individual QUADAS items and additional variables thought likely to be associated with diagnostic accuracy were associated with the DOR and hence whether differences in these items between the studies accounted for some of the observed heterogeneity. Where data were available, the following additional variables were investigated:

• Patient age (<2 years, <5 years, <12 years and <18 years) was included to examine possible variation with age within the paediatric population.

• The geographic region where studies were conducted was included to account for possible regional differences in test technology and infective agent.

• Specific variations in index test technique were also included. For microscopy for pyuria and bacteriuria a variable on whether the sample was centrifuged was included, and for microscopy for bacteriuria a variable for Gram stain was included. For ultrasound for the detection of reflux a variable for whether or not the ultrasound involved a contrast agent was included.

• The SROC model [15], was extended to include each of the 14 QUADAS items and each of the variables above as individual covariates [17]. As each QUADAS item can be scored as "yes", "no" or "unclear", we included QUADAS items as categorical variables with 3 possible outcomes, thus including the comparisons of "yes vs no", and "yes vs unclear". This allowed us to make some distinction between associations of aspects of methodological quality with test performance and associations of completeness of reporting with test performance. A number of QUADAS items only received two of the three possible scores (i.e. were scored either "yes" or "no", or "yes" or "unclear", or "no" or "unclear"). These items were therefore included as dichotomous variables.

A multivariate linear regression analysis was conducted. Initially, we performed univariate analysis with all items included separately in the model. Items that showed moderate evidence of an association with D, defined as p < 0.10, were investigated further using step-down regression analysis. All items found to show moderate evidence of an association in the univariate models were entered into the multivariate model, then dropped in a step-wise fashion with the item with the weakest evidence of an association (largest p-value) dropped first. For covariates with more than one level, evidence of an association of one indicator variable with test performance was considered sufficient for inclusion in the model. The final model was achieved when all items remaining showed strong evidence of an association with D, defined as p < 0.05. Interaction terms were not included. Associations of covariates with D were expressed as relative diagnostic odds ratios (RDOR). The DOR is used as an overall measure of diagnostic accuracy. It is calculated as the odds of positivity among diseased persons, divided by the odds of positivity among non-diseased. When a test provides no diagnostic evidence then the DOR is 1.0. The RDOR is calculated as the DOR when the covariate is present divided by the DOR when the covariate is absent. It therefore provides an indicator of the overall impact on diagnostic accuracy of the presence of a given covariate.

## Results

### Results of QUADAS assessment

**Figure 1:**
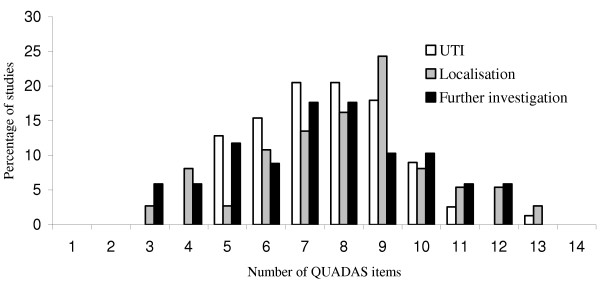
Numbers of quality items fulfilled by studies in the three sections of the review.

The proportion of QUADAS items fulfilled by studies included in our systematic review was similar for each of the three clinical stages assessed in the review. Studies evaluating tests to diagnose UTI fulfilled a median of 8 (range 5–13) items, those evaluating tests used to localise infection also fulfilled a median of 8 (range 3–13) items, and those evaluating further investigations fulfilled a median of 7.5 (range 3–12) items. Figure 1 illustrates the number of QUADAS items fulfilled by studies in each category. The similarity in numbers of QUADAS items fulfilled masks apparent differences in the individual items fulfilled.

**Figure 2:**
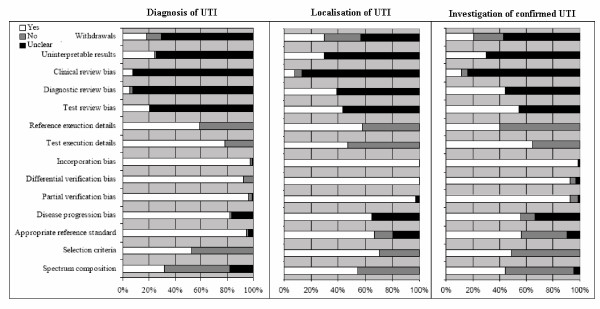
Proportion of studies rated as yes, no or unclear for each of the QUADAS items, separately for diagnosis of UTI, localisation of infection and investigation of confirmed UTI.

Figure 2 shows the proportion of studies that scored "yes", "no" and "unclear" for each of the QUADAS items, separately for the three sections of the review.

#### Tests for the diagnosis of UTI (n = 79 studies) [18-96]

The use of an inappropriate spectrum of patients and inadequate reporting of inclusion criteria were problematic for studies in this category. The majority of studies provided insufficient details on how the reference standard was performed. Studies failed to report sufficient details on clinical review bias, diagnostic review bias and test review bias to judge whether these were avoided. Study withdrawals and handling of uninterpretable results were also poorly reported.

#### Tests for the localisation of infection (n = 39 studies) [48,69,97-133]

The time delay between the index test and reference standard was more of a problem with these studies than with those on the diagnosis of UTI. The use of an appropriate reference standard was also an issue in some of these studies. Spectrum composition and reporting of details of how children were selected for inclusion in the study was better in these studies than in the studies of the diagnosis of UTI. Only around half of studies provided sufficient details of how the index test and reference standard were performed to allow replication of these tests. More studies in this category, almost 40%, provided information indicating that test and diagnostic review bias had been avoided, in the remainder of studies this information was not reported. As with studies of the diagnosis of UTI, reporting of clinical review bias, handling of uninterpretable results, and withdrawals from the study was poor.

#### Tests for the further investigation of confirmed UTI (n = 71 studies) [134-204]

As with studies of the diagnosis of UTI, spectrum composition and reporting of inclusion criteria were poor in this group. The time delay between the index test and reference standard was also an issue in many of these studies. Around half of studies reported that diagnostic and test review bias had been avoided, the remaining studies did not report whether the index test and reference standard were interpreted blind to the results of each other. This was similar to the situation seen for studies on the localisation of infection. Reporting of the reference standard was poor. As in all previous groups, studies also provided very little information on whether appropriate clinical information was available when test results were interpreted, how uninterpretable results were handled, and whether there were any withdrawals from the study and if so whether all withdrawals were accounted for.

### Results of multivariate regression analysis

#### Tests for the diagnosis of UTI

Tests involving dipstick or microscopy techniques were the only categories where enough studies were available to enable regression analysis. Table 3 summarises the results of the regression analysis for studies assessing dipstick tests. For dipstick to detect urinary nitrite (23 studies) [20,26,28,34,36,40,41,43,52,54-57,60,63,66,72,74,84,88,93-95], the weighted analysis found that studies reporting that clinical review bias had been avoided had a DOR 4.7 (95% CI: 1.7, 12.7) times greater than those which did not report on whether clinical information was available to those interpreting the test results (p = 0.004). This is what would be expected, as the DOR is likely to be higher when those interpreting test results have access to appropriate clinical information similar to that, which would be available in practice. No studies reported the presence of clinical review bias. This was the only item investigated to show strong evidence of an association with test performance in the weighted multivariate analysis, although age and geographic region did show moderate evidence of an association in the univariate analysis. The unweighted analysis showed slightly different results. The same three items were found to show at least moderate evidence of an association in the univariate analysis. However, only country remained in the multivariate model, suggesting that studies conducted in North America showed higher accuracy than studies conducted in Europe or other areas (p < 0.05).

**Table 3:** Results of the regression analysis for dipstick tests for the diagnosis of UTI

	**Univariate analysis**		**Multivariate model**	
	
Variable*	**RDOR (95% CI)**	**p-value**	**RDOR (95% CI)**	**p-value**
**Nitrite dipstick – weighted (n = 23 studies)**				

Clinical review bias avoided: yes vs unclear	4.7 (1.7, 12.7)	0.004	4.7 (1.7, 12.7)	0.004
Age <5 years vs <2 years	5.8 (0.3, 101.8)	0.213	Dropped^$^	
Age <12 years vs <2 years	2.2 (0.1, 35.8)	0.548	Dropped	
Age <18 years vs <2 years	3.6 (0.9, 14.9)	0.076	Dropped	
Europe vs North America	0.3 (0.1,0.9)	0.041	Dropped	
Other areas vs North America	0.3 (0.1, 1.0)	0.050	Dropped	

Nitrite dipstick – unweighted (n = 23 studies)				

Clinical review bias avoided: yes vs unclear	3.4 (0.9, 13.7)	0.078	Dropped	
Age <5 years vs <2 years	7.3 (0.9, 61.8)	0.067	Dropped	
Age <12 years vs <2 years	2.9 (0.5, 18.3)	0.243	Dropped	
Age <18 years vs <2 years	3.8 (1.1, 13.4)	0.039	Dropped	
Europe vs North America	0.3 (0.1, 1.0)	0.044	0.3 (0.1, 1.0)	0.044
Other areas vs North America	0.3 (0.1, 1.2)	0.089	0.3 (0.1, 1.2)	0.089

LE dipstick – weighted (n = 14 studies)				

No association at p < 0.10				

LE dipstick – unweighted (n = 14 studies)				

Test details reported: yes vs no	19.0 (1.9, 192.2)	0.017	Dropped	
Age <5 years vs <2 years	5.4 (0.5, 64.2)	0.158	5.4 (0.5, 64.2)	0.158
Age <12 years vs <2 years	28.1 (2.3, 343.3)	0.015	28.1 (2.3, 343.3)	0.015
Age <18 years vs <2 years	1.3 (0.3, 4.7)	0.703	1.3 (0.3, 4.7)	0.703

Nitrite or leukocyte esterase dipstick – weighted (n = 15 studies)				

Reference standard details reported	4.5 (0.9, 22.5)	0.064	Dropped	
North America vs Europe	5.0 (0.8, 10.5)	0.076	Dropped	
North America vs Other areas	1.1 (0.28, 5.0)	0.854	Dropped	

Nitrite or leukocyte esterase dipstick – unweighted (n = 15 studies)				

No association at p < 0.10				

*Only items that showed moderate evidence (p < 0.10) for an association with the DOR in the univariate analysis are included.

^$^Items were dropped where there were too few studies in one category to allow a coefficient to be calculated.

For dipsticks measuring urinary leukocyte esterase (14 studies) [20,28,34,36,43,56,57,60,63,66,72,84,94,95] and for dipsticks for the presence of either nitrite or leukocyte esterase (15 studies) [19-21,28,34,56,60,63,66,84-86,92,94-96], no items showed strong evidence of an association with the DOR in the weighted analysis. However, for urinary leukocyte esterase, the unweighted analysis found strong evidence of an association between patient age and the DOR. There was strong evidence (p = 0.015) that the dipstick was more accurate in children aged <12 years than in those aged <2 years (RDOR = 28.1, 95% CI: 2.3, 343.3). There was no evidence of any difference in accuracy between children aged <18 years and those aged <2 years (p = 0.703), and very little evidence of any difference between children aged <5 years and those aged <2 years (p = 0.158).

Table 4 summarises the results of the regression analysis for studies that assessed the accuracy of microscopy. In studies evaluating microscopy to detect pyuria three items showed a strong association with test performance in the weighted analysis (28 studies) [19-23,28,29,34,35,41,43,46,47,49,50,58,59,63,67,70,75,77,80,81,83,85,92-94]. The DOR was 1.3 (95% CI: 1.1, 1.6; p = 0.007) times higher in studies that adequately reported details of the reference standard execution. The DOR was lower, RDOR = 0.2 (95% CI: 0.1, 0.4; p < 0.001) in studies that did not report on reasons for withdrawals compared to studies in which it was unclear whether there were any withdrawals, and 1.8 times higher (95 % CI: 1.0, 3.4; p = 0.056) in studies in which withdrawals were accounted for compared to those in which this was unclear. The DOR was lower, RDOR = 0.2 (95% CI: 0.1, 0.3; p < 0.001), in studies where samples were centrifuged compared to studies in which samples were not centrifuged. In the unweighted analysis, only centrifugation showed any evidence of an association with test performance (p = 0.08). All of these items, with the exception of centrifugation, relate to the completeness of reporting. The association for centrifugation is counter-intuitive, as we would expect centrifugation of the sample to lead to improved test accuracy.

**Table 4:** Results of the regression analysis for microscopy for the diagnosis of UTI

	**Univariate analysis**		**Multivariate model**	
	
Variable*	**RDOR (95% CI)**	**p-value**	**RDOR (95% CI)**	**p-value**
**Microscopy for pyuria: weighted (n = 28 studies)**				

Selection criteria reported: yes vs no	2.4 (1.0, 5.9)	0.057	Dropped^$^	
Reference standard details reported: yes vs no	3.6 (0.8, 16.1)	0.089	1.3 (1.1, 1.6)	0.007
Test review bias avoided: yes vs unclear	4.8 (1.6, 14.7)	0.008	Dropped	
Diagnostic review bias avoided: yes vs unclear	5.5 (1.8, 17.1)	0.005	Dropped	
Uninterpretable results reported: no vs unclear	0.3 (0.0, 4.1)	0.364	Dropped	
Uninterpretable results reported: yes vs unclear	2.9 (0.9, 9.1)	0.073	Dropped	
Withdrawals accounted for: no vs unclear	0.2 (0.1, 0.7)	0.012	0.2 (0.1, 0.4)	<0.001
Withdrawals accounted for: yes vs unclear	1.9 (0.7, 5.3)	0.200	1.8 (1.0, 3.4)	0.056
Europe vs North America	0.3 (0.1, 1.6)	0.190	Dropped	
Asia vs North America	0.6 (0.1, 6.6)	0.646	Dropped	
Other areas vs North America	0.2 (0.0, 1.3)	0.090	Dropped	
Age <5 years vs <2 years	0.1 (0.0, 0.4)	0.004	Dropped	
Age <12 years vs <2 years	0.1 (0.0, 0.3)	0.004	Dropped	
Age <18 years vs <2 years	0.2 (0.1, 0.5)	0.001	Dropped	
Sample centrifuged: yes vs no	0.3 (0.1, 0.6)	0.005	0.2 (0.1, 0.3)	<0.001

**Microscopy for pyuria: unweighted (n = 28 studies)**				

Age <5 years vs <2 years	0.3 (0.1, 1.1)	0.076	Dropped	
Age <12 years vs <2 years	0.5 (0.1, 2.1)	0.337	Dropped	
Age <18 years vs <2 years	0.3 (0.1, 1.0)	0.048	Dropped	
Sample centrifuged	0.5 (0.2, 1.1)	0.080	0.5 (0.2, 1.1)	0.080

**Microscopy for bacteriuria: weighted (n = 22 studies)**				

Incorporation bias avoided: no vs yes	24.5 (1.0, 604.4)	0.050	3.0 (1.6, 5.5)	0.001
Diagnostic review bias avoided: yes vs unclear	3.2 (0.8, 12.8)	0.092	Dropped	
Gram stain used: yes vs no	3.6 (1.3, 10.4)	0.018	5.3 (2.3, 12.0)	0.001

**Microscopy for bacteriuria: unweighted (n = 22 studies)**				

Disease progression bias: yes vs unclear	0.05 (0.0, 1.5)	0.083	Dropped	
Incorporation bias avoided: no vs yes	32.5 (1.2, 895.0)	0.041	3.2 (1.6, 6.4)	0.003
Uninterpretable results reported: yes vs unclear	7.1 (1.1, 46.9)	0.042	Dropped	

Withdrawals reported: no vs unclear	6.6 (1.0, 43.3)	0.049	Dropped	
Withdrawals reported: yes vs unclear	2.2 (0.3, 18.7)	0.447	Dropped	
Sample centrifuged: yes vs no	0.2 (0.0, 1.1)	0.058	Dropped	
Gram stain used: yes vs no	3.9 (0.9, 16.3)	0.062	6.5 (2.0, 21.2)	0.004

*Only items that showed moderate evidence (p < 0.10) for an association with the DOR in the univariate analysis are included.

^$^Items were dropped where there were too few studies in one category to allow a coefficient to be calculated.

Two items showed a strong evidence of an association with the DOR in the weighted analysis of studies evaluating microscopy to detect bacteriuria (22 studies) [20,21,23,28,34,35,41,47,50,61-64,67,70,76,77,80,85,90,91,94]. The DOR was 3.0 (95% CI: 1.6, 5.5, p = 001) times greater in studies in which incorporation bias was present compared to those in which it was avoided, and 5.3 (95% CI: 2.3, 12.0, p = 0.001) times greater if samples were Gram stained. We would expect both Gram staining and the presence of incorporation bias to increase test performance as found in the analysis. The unweighted analysis found very similar results.

#### Tests for the localisation of infection

Only the evaluation of ultrasound for the localisation of infection provided sufficient data to enable the conduct of regression analysis (20 studies) [48,69,97,99-102,109-111,113-115,117,118,121,126,128,132,133]. None of the QUADAS items, or other items investigated, showed moderate evidence of an association with the DOR in this analysis, using either the weighted or unweighted model.

#### Tests for the further investigation of confirmed UTI

Table 5 summarises the results of the regression analysis for studies assessing this clinical stage. The use of ultrasound to detect reflux was the only test in this category with sufficient data to support regression analysis (28 studies) [69,135,137,140,141,150,152,153,155,164,169,170,172,176-178,181,185,187,189,190,195-198,202-204]. Three items showed strong evidence of an association with the DOR in the weighted analysis. The DOR was 8.0 (95% CI: 2.9, 22.0; p < 0.001) times greater in studies that used contrast enhanced ultrasound compared to those that used standard ultrasound. As this was also thought to be a clinically important variable it was included in all further analyses. The DOR was 1.4 (95% CI: 1.0, 1.9; p = 0.033) times higher in studies that reported that disease progression bias had been avoided compared to those in which this information was not reported. No studies reported sufficient information to determine that disease progression bias was present. Studies in which details were provided on reasons for withdrawals had DORs that were 2.8 times higher (95% CI: 1.1, 6.9, p = 0.033) than those in which it was unclear whether there had been any withdrawals. There was no evidence of any difference in the DOR between studies that did not report on reasons for withdrawals and studies in which it was unclear whether there were any withdrawals (p = 0.502). In the unweighted analysis, only two items showed a strong evidence of an association with the DOR. As in the weighted analysis there was very strong evidence that the DOR was higher in studies that used contrast enhanced ultrasound than those that used standard ultrasound (RDOR = 29.8, 95% CI: 13.5, 65.8, p < 0.001). Studies in which partial verification bias was avoided had DORs 4.1 times higher (95% CI: 1.1, 14.8) than those that did not (p = 0.034).

**Table 5:** Results of the regression analysis for ultrasound for the diagnosis of reflux

	**Univariate analysis**		**Multivariate analyis**	
	
Variable*	**RDOR (95% CI)**	**p-value**	**RDOR (95% CI)**	**p-value**
**Ultrasound for the detection of reflux: weighted (n = 28 studies)**				

Use of contrast enhanced ultrasound: yes vs no	23.9 (9.8, 58.8)	<0.001	8.0 (2.9, 22.0)	<0.001

**Ultrasound for the detection of reflux, with ultrasound type forced into the model: weighted (n = 28 studies)**				

Appropriate reference standard: yes vs unclear ^+^	0.2 (0.0, 1.0)	0.047	Dropped^$^	
Disease progression bias avoided: yes vs unclear	3.5 (1.4, 9.2)	0.011	1.4 (1.0, 1.9)	0.033
Withdrawals accounted for: yes vs unclear	3.2 (1.2, 8.5)	0.020	2.8 (1.1, 6.9)	0.027
Withdrawals accounted for: no vs unclear	(0.4, 0.1, 1.7)	0.175	0.6 (0.1, 2.8)	0.502

**Ultrasound for the detection of reflux: unweighted (n = 28 studies)**				

Use of contrast enhanced ultrasound: yes vs no	29.8 (13.5, 65.8)	<0.001	29.8 (13.5, 65.8)	<0.001

**Ultrasound for the detection of reflux, with ultrasound type forced into the model: unweighted (n = 28 studies)**				

Appropriate reference standard *: yes vs unclear	0.2 (0.0, 1.2)	0.075	Dropped	
Partial verification bias avoided: yes vs no	4.1 (1.1, 14.8)	0.034	4.1 (1.1, 14.8)	0.034

*Only items that showed moderate evidence (p < 0.10) for an association with the DOR in the univariate analysis are included.

^+^Only 1 unclear

^$^Items were dropped where there were too few studies in one category to allow a coefficient to be calculated.

## Discussion

The methodological quality of primary studies remains a significant issue for systematic reviews of diagnostic tests [8,205,206]. The STARD initiative has provided clear guidance for the reporting of diagnostic accuracy studies [5]. This should have a positive impact on the quality of the diagnostic literature in the future. The QUADAS tool facilitates systematic evaluation of the quality of diagnostic accuracy studies, and was specifically developed for use in systematic reviews of diagnostic tests [12]. However, where studies are poorly reported the information that can be derived from quality assessment becomes limited. We cannot know whether an unreported QUADAS item reflects a true methodological flaw or poor reporting of a study that may be methodologically sound. Many of the studies included in our review were poorly reported. Our assessment of the impact of components of methodological quality on diagnostic accuracy may therefore partially reflect completeness of reporting. Whilst poor reporting remains a widespread problem, it is almost impossible to assess the impact of components of methodological quality on the results of diagnostic meta-analyses.

The common practice of using summary quality scores in systematic reviews has been widely debated elsewhere [207-209]. Summary scores, when used to inform quality-based analyses, may mask important effects of individual quality components [210]. As we report, the numbers of QUADAS items that were adequately addressed by studies included in our review were similar between the three clinical stages assessed in the review. Had the number of QUADAS items fulfilled been used as a summary score, potentially important variations in the individual items fulfilled would have been hidden. We therefore advocate that components of quality assessment should be reported fully, and their impact on outcome measures analysed individually rather than as summary scores.

Although ours was a large review, it included 187 studies reporting 487 data sets, our analysis of the impact of methodological quality on diagnostic accuracy was severely limited both by the diversity of the included studies (few tests were evaluated by sufficient studies to allow meaningful use of meta-analytic pooling and investigation of heterogeneity), and by incomplete reporting. All of the data sets used were sub-optimal, in that the numbers of observations were low in comparison to the number of variables investigated in the multivariate analyses[13]. Although different types of diagnostic tests were evaluated in the three clinical stages used by the review, generalisibility is limited in that all data concerned a single condition (UTI). A number of the items found to be associated with test performance related to specific test methodologies (e.g. Gram stain and contrast-enhanced ultrasound) and have no generalisability elsewhere. These items were found to show association in both the weighted and unweighted analyses. For the individual quality items there were some differences between the results of the weighted and unweighted analyses. In general, the results of weighted analyses showed more intuitive associations. Unweighted analyses more often produced results that were difficult to explain, for example, in leukocyte esterase dipstick tests the unweighted analysis found that the test was more accurate in the group of children aged <12 years than in those aged <2 years. This might be expected and would probably reflect a higher likelihood of sample contamination in younger children, however, no difference in accuracy was found between under 18's and children aged <2 years. For both tests on the diagnosis of and further investigation of UTI weighted analyses showed an association between a number of variables relating to quality of reporting and diagnostic accuracy (well reported studies had higher DORs). We might expect this association to extend to diagnostic accuracy studies of all types of tests, but the present study is not adequate to demonstrate this. Weighted analysis of studies of ultrasound for the detection of reflux showed that the DOR was higher where studies reported information to determine that disease progression bias had been avoided. Disease progression bias is a particular issue for imaging studies of this type where follow-up examinations (used as the reference standard of diagnosis) may be scheduled some time after ultrasound (usually the initial examination). This association was not shown in the unweighted analysis.

The information derived from these analyses is also limited by the use of the summary ROC approach to pool studies. This method takes the DOR as the dependent variable. The DOR is used as a single indicator of test performance and shows how much more frequently a positive test result occurs in a person with the condition of interest than in one without the condition, relative to how much more frequently a negative result occurs in a person without the condition than in one with the condition. Using the DOR to investigate heterogeneity means that we cannot assess whether the factors investigated are associated with paired measures of diagnostic accuracy, such as sensitivity and specificity, or positive and negative likelihood ratios. Often factors that lead to an increase in sensitivity will lead to a decrease in specificity and vice versa. Factors that lead to this pattern of change may have no effect on an overall measure such as the DOR. Using the DOR to investigate heterogeneity may thus miss relevant clinical associations. Recently a new method for pooling sensitivity and specificity has been developed. This method is known as the "bivariate model" [211]. It preserves the underlying two-dimensional nature of the data and produces direct pooled estimates of sensitivity and specificity, incorporating any correlation that might exist between these two measures. The model can be extended to include explanatory variables leading to separate effects on sensitivity and specificity. This method has two advantages over the standard methods: (1) the pooled estimates of sensitivity and specificity take into account the correlation between these two measures; (2) the effect of possible sources of heterogeneity on both sensitivity and specificity can be investigated in a single model rather than just looking at the effect of these variables on a single measure of test performance, the DOR. These methods may have potential applications in future studies of this type.

## Conclusion

Given the limitations we describe, the results of this study should be treated as hypothesis generating. Further work is needed to elucidate the influence of components of the methodological quality of primary studies on the results of diagnostic meta-analyses. Large data sets of well-reported primary studies are needed to address this question. Without significant improvements in the reporting of primary studies, progress in this area will be limited. The components of quality assessment should always be reported, and their impact on summary outcome measures be investigated, individually rather than as summary quality scores. Careful consideration should be given to the choice of weighting when conducting regression analyses. Weighting by sample size appears the most appropriate method for analyses of diagnostic accuracy studies, but this area requires further investigation.

## Competing interests

The author(s) declare that they have no competing interests

## Authors' contributions

All authors contributed towards the conception and design of the study and the interpretation of the data. They also read and approved the final manuscript. PW and MW participated in data extraction, the analysis of data, and drafted the article.

## Pre-publication history

The pre-publication history for this paper can be accessed here:


